# Multivariate analysis reveals phenotypic diversity of *Euscaphis japonica* population

**DOI:** 10.1371/journal.pone.0219046

**Published:** 2019-07-16

**Authors:** Weihong Sun, Xueyan Yuan, Zhong-Jian Liu, Siren Lan, Wen-chieh Tsai, Shuang-Quan Zou

**Affiliations:** 1 College of Forestry, Fujian Agriculture and Forestry University, Fuzhou, China; 2 Fujian Colleges and Universities Engineering Research Institute of Conservation and Utilization of Natural Bioresources, College of Forestry, Fujian Agriculture and Forestry University, Fuzhou, China; 3 Key Laboratory of National Forestry and Grassland Administration for Orchid Conservation and Utilization at Colleage of Landscape Architecture, College of Landscape Architecture, Fujian Agriculture and Forestry University, Fuzhou, China; 4 Ornamental Plant Germplasm Resources Innovation and Engineering Application Research Center, College of Landscape Architecture, Fujian Agriculture and Forestry University, Fuzhou, China; 5 Department of Biological Sciences and Technology, National University of Tainan, Tainan, Taiwan, China; Peking University, CHINA

## Abstract

Fruit traits affect population genetic diversity by affecting seed protection and dispersal strategies, thereby comprising important components of phenotypic variation. Understanding of the phenotypic variation is an indispensable first step for developing breeding strategies. However, little information is known about the genetic variation in *E*. *japonica*—a monotypic species with abundant phenotypes that is mainly distributed in southern China. In this study, we evaluated the phenotypic diversity of 67 *E*. *japonica* using 23 phenotypic traits. Our results showed that the Shannon–Wiener (*I*) index of qualitative traits ranged from 0.55 to 1.26, and the color traits had a relatively high *I*. The average coefficient of variation of compound leaf traits (14.74%) was higher than that of fruit and seed traits (12.77% and 11.47%, respectively). Principal component analysis also showed that compound leaf and fruit traits were important components of total variation. Furthermore, correlation analysis revealed a significant difference in elevation and fruit color, irregular ribs, leaf margin and texture. The F value within populations was smaller than among populations, indicating the variation in phenotypic traits among populations was much greater than within populations. Dehua and Zunyi populations had the highest coefficients of variation, whereas Wenzhou population had the smallest—which may be attributed to habitat destruction. According to Q-type clustering, 67 samples clustered into four groups, with those having similar phenotypes clustering into the same group. In general, leaf and fruit traits had abundant phenotypic diversity, representing the main sources of phenotypic variation. Combined with clustering results and field surveys, this study suggests that the phenotypes of *E*. *japonica* are classified into two main categories: The deciduous *E*. *japonica* present at high altitudes; and the evergreen *E*. *japonica* present at low altitudes. Excavating *E*. *japonica* variations provides a theoretical reference for its classification and diversity, and is of great significance for planning genetic resources and establishing conservation strategies.

## Introduction

Tree evolution is largely driven by adapting previous seed protection and dispersal strategies that allow diversification into new niches [1]. Seed maturity and dispersion are inextricably linked to fruit type, e.g., dry fruit is mainly used to help seed dispersal by cracking. In most trees, fruit colors are also crucial for attracting seed-dispersing organisms (such as squirrel and birds), and for both commercial and ornamental value [2,3]. Therefore, variations in fruit traits are very important aspects of a population’s survival and genetic diversity during evolution.

Genetic diversity, a fundamental source of biodiversity, provides the raw material for evolution by natural selection [4], which includes phenotype, protein, and DNA variations. Phenotypic variations are highly recommended as a first step prior to attempting more in-depth biochemical or molecular studies [5]. Hence, morphological description remains the first step in the process of plant genetic diversity preservation [6]. Using variation traits to study phenotypic diversity, revealing the genetic structure and variation size of the population form the basis of genetic breeding. Phenotypic variance is thought to be the result of natural selection, reflecting both adaption to local environmental characteristics and genetic diversity [7–9], such as meteorological change, trait mutation, and genetic drift [10]. Related studies have confirmed that phenotypic plasticity is a major means by which plants’ cope with environmental factor variability [11]. Phenotypic plasticity can evolve when sufficient genetic variation is present [12,13], either due to genetic correlations with other traits that are under selection or genetic drift [14]. Thus, plant phenotypes result from interactions between genotype and environment, reflecting genotype adaptation to environmental changes. Phenotypes are formed as a result of long-term stress selection, and represent irreversible processes that can be stably inherited by offsprings. Phenotypes reflect plants’ environmental adaptability, and thus phenotypic variation is of great significance in adaptation and classification.

*Euscaphis* (Staphyleaceae) consists of one monotypic species, *Euscaphis japonica*, only distributed in southern China, Japan, and Korea. It is found as populations of scattered individuals, associated with forests and broad- leaved species in streams or valleys [15,16]. According to Flora of China records [15], *E*. *japonica* is a deciduous tree or shrub, flowering from April to June, and fruiting from August to December. However, during long-term field observations, we found that *E*. *japonica* are a deciduous or evergreen tree with significant phenotypic differences at varying altitudes, especially with respect to leaves and fruit. In addition, it has been previously discovered that *E*. *japonica* has existed on Earth for 33.9 million years [17], which inspired our curiosity as to why *Euscaphis* has one species only despite its wide range of distribution and long survival time. Therefore, some causes of phenotypic diversity in *E*. *japonica* could be explained by exploring phenotypic variation.

Since ancient times fruit from *E*. *japonica* have been used as valuable medicinal material for people in southern China to treat headaches, dizziness, colds, urticaria, hernia, and rheumatism [18–21]. In recent years, several chemical compounds have been isolated from *E*. *japonica*, such as triterpene [22,23], phenolic acids [24, 25], flavonoids [8, 22], etc. [26,27]. In addition, *E*. *japonica* is an excellent ornamental tree species for its unique fruit-shaped and red pericarp. This tree has been cultivated on a large scale as an ornamental and medicinal tree species in Fuzhou, Jiangxi and Guizhou Provinces, in southern China. However, the economic potential of its cultivation has been poorly addressed. Thus, *E*. *japonica* is considered to be an underutilized species in this region, mainly due to the limited information on *E*. *japonica’s* genetic structure and phenotype characterization, as well as the lack of growing guidelines for producers. Therefore, a strategy for evaluating and conserving genetic resources is necessary for preserving the existing genetic variability of *E*. *japonica* in China. In the current study, the main objectives were the use of phenotypic characters (i) to reveal the degree of phenotypic variation among and within populations, (ii) to explore the correlation between phenotypic traits and their geographical ecology, and (iii) to provide a theoretical reference for the classification, diversity, and conservation *E*. *japonica*-related studies.

## Materials and methods

### Plant material

The field collection was conducted from 2016 to 2017 in southern China during the fruiting period of *E*. *Japonica*. The number *E*. *japonica* populations are present in small areas of natural communities. We sampled the individuals with fruit found and recorded the latitude, longitude, altitude, and specific habitat of populations (Table 1). Sixty-seven samples from eight populations were studied.

**Table 1:** The collecting place, traits and habitat of *E*. *japonica*.

population (size)	location	code	longitude	latitude	altitude /m	evergreen/ deciduous	leaf margin	leaf texture	fruit epidermis rib	habitat
Zunyi (9)	Guizhou Xishui National Nature Reserve, Zunyyi, Guizhou Province	ZYY1~ZYY9	106°40′	28°49′	918	deciduous	serrulate	papery	prominent	Locate at the forest-edge or the shrubbery by the side of stream.
Wenzhou (5)	Dailing Mountain, Wenzhou, Zhejiang Province	WZY1~WZY5	120°24′	27°16′	314	deciduous	serrulate	papery	prominent	Trees high 1.5~4.5m, locate in the valley and cliffs.
Jiangxi (13)	Ganzhou, Jiangxi Province	JX1~JX13	114°55′	25°23′	151	evergreen	obtuse serrate	membranous	inconspicuous	Tree high 3~7m, locate at the jungle, forest margin
Qingliu (10)	Lingdi Tomn, Sanming, Fuzhou Province	QL1~OL10	116°48′	25°50′	359	evergreen	obtuse serrate	membranous	inconspicuous	Tree high 7~12 m, each tree is at least 100 years old and transplanted from the local forest to The Company of Yisheng Agriculture and Forestry.
Taining (5)	Meikou Tomnship, Sanming, Fuzhou Province	TN1~TN5	117°06′	27°48′	302	evergreen	obtuse serrate	membranous	inconspicuous	Tree high 3~5 m, locate at the side of the road and hillside.
Jianyang (3)	Songbai Township, Jianyan, Nanping, Fuzhou Province	JY1~JY3	118°02′	27°26′	174	evergreen	obtuse serrate	membranous	inconspicuous	Tree high 3~5 m, locate at the valley
Jianou (3)	Jianou, Nanping, Fuzhou Province	JO1~JO3	118°18′	27°00′	195	evergreen	obtuse serrate	membranous	inconspicuous	Tree high 2~4 m, locate at the valley.
Jiangshi (13)	Jiangshi Nature Reserve, Shaowu, Fujian Province	JS1~JS13	117°14′	27°03′	408	evergreen	obtuse serrate	membranous	inconspicuous	Tree high 4~6 m, locate in the valley、forest margin and secondary forest.
Dehua (6)	Gaoyang village, Quanzhou, Fuzhou Province	DH1~DH3	118°14′	25°29′	494	evergreen	obtuse serrate	membranous	inconspicuous	Tree high 10~15 m, each tree is at least 100 years old, locate in the bamboo forest by the stream.
	Shiliu Mountain, Quanzhou, Fuzhou Province	DHY1~DHY3	118°29′	25°42′	1000	deciduous	serrulate	thick paper	prominent	DHY1 is at least 100 years old and high 8 m, DHY2 and DHY3 high 1.5 m and 2.4 m, respectively. Locate in the bamboo forest by the stream.

The sampling work of Zunyi and Jiangshi poopulations were permitted and approved by Guizhou Xishui Nature Reserve and Jiangshi Nature Reserve respectively; The sampling work of Dehua population was permitted and approved by Quangzhou Forestry Bureau; The sampling work of Taining population was permitted and approved by Sanming Forestry Bureau; The sampling work of Jianyang and Jianou were permitted and approved by Nanping Forestry Bureau; The sampling work of Qingliu population was permitted and approved by the Company of Yisheng Agriculture and Forestry, which land accessed is privately owned. Ou Bin (College of Forestry, Jiangxi Environmental Engineering Vocational College, Jiangxi Province) and Zou Zhuang (Zhejiang Academy of Agricultural Sciences) assist us to collect samples from Jiangxi and Wenzhou population. Furthermore, *E*.*japonica* does not belong to the national protected tree species. We only collect young leaves,so there is no damage to the trees.

### Phenotypic evaluation

**Fig 1:**
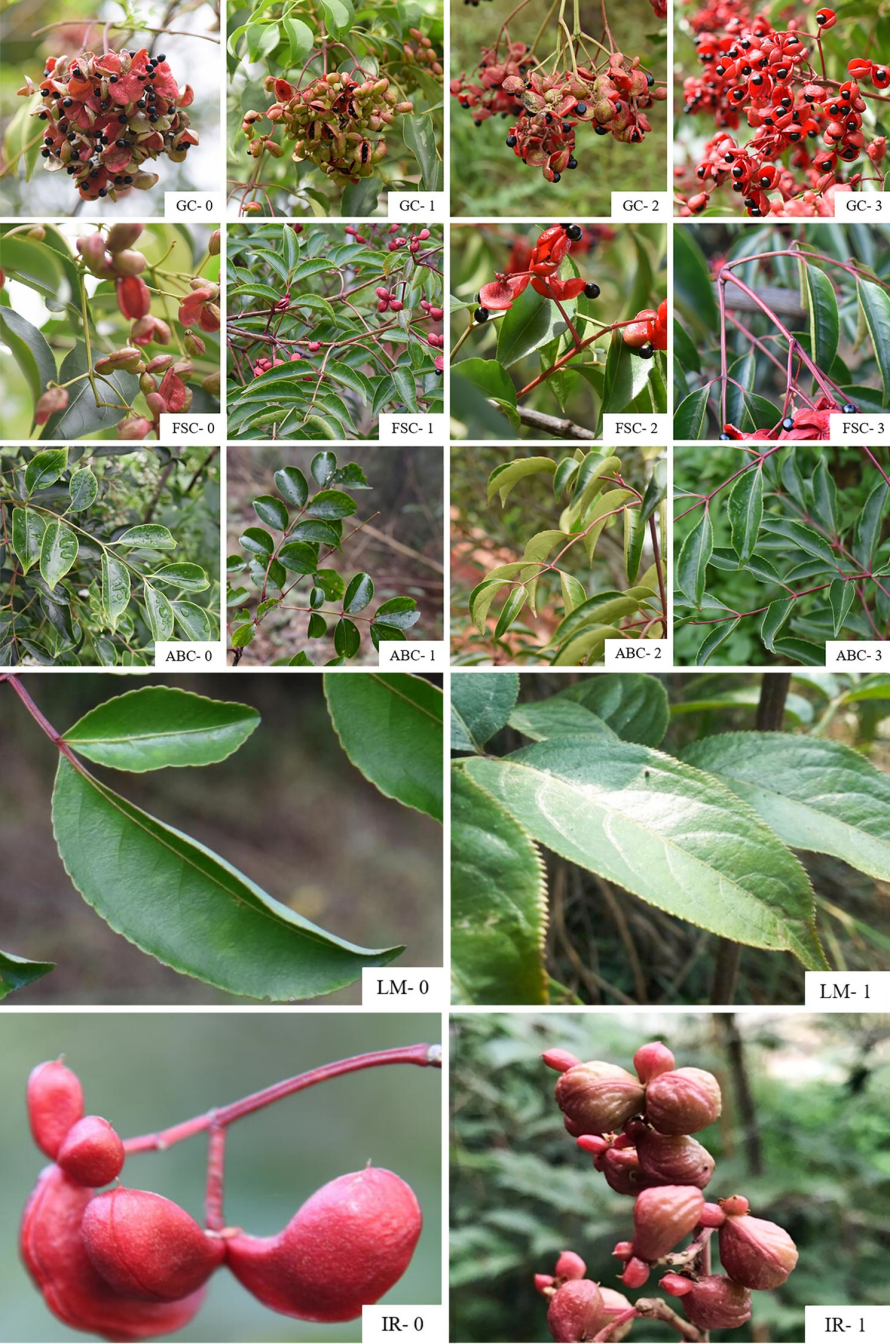
Different phenotypic traits of *E japonica* leaves and fruits. The coding of relevant colors (fruit color, fruit sequence color, and compound petiole color) was divided into four types, and encoded by the ratio of green and red. 0-type: Green/red = 2; 1-type: Green/red = 1; 2-type: Green/red = 1/2; 3-type: Red; LM-0: leaf margin obtuse serrate, LM-1: Leaf margin serrulate; IR-0: Fruit epidermis irregular ribs inconspicuous, IR-1: Epidermis ribs prominent.

Through the observation of the flower morphology of different *E*. *japonica* populations, we found that the flower morphology was stable, including color, size, etc. Therefore, the flower characteristics were not discussed in this paper. Twenty-three phenotypic traits, both quantitative and qualitative, were measured (Table 2). Since the color traits (such as: compound petiole color; annual branch color; fruit color; fruit sequence color) hardly change after the fruit cracks, therefore, the original records of qualitative traits were obtained from field observation in November 2016 and 2017. The obtained original record was then converted into a form suitable for mathematical operations according to Table 2. There are seven important qualitative traits and corresponding codes (Fig 1). The relevant color codes were divided into four types, encoded by the ratio of green and red (Table 2; Fig 1). Sixteen quantitative characters were present, and the measured value or count value was coded. Fruit width, fruit length, pericarp thickness, seed length, seed width, and petiole length were measured using Vernier calipers (precision 0.01mm). Compound leaf length, leaflet length, and leaf width were measured using a ruler (precision 0.1cm).

**Table 2:** The code of morphologies of *E*. *japonica*.

No.	Traits	Encoded	No.	Traits	Encoded
1	Compound leaf length (CLL)		13	Fruit width (FW)	
2	Compound petiole color (CPC)	green/red = 2 0; green/red = 1 1; green/ren = 1/2 2; red 3	14	Fruit length (FL)	
3	Annual branch color (ABC)	green/red = 2 0; green/red = 1 1; green/red = 1/2 2; red 3	15	Fruit index (FI)	
4	Leaflet number (LN)		16	Fruit color (FC)	green/red = 2 0; green/red = 1 1; green/red = 1/2 2; red 3
5	Leaflet area (LA)		17	Pericarp thickness (PT)	
6	Leaflet circumference (LC)		17	Irregular ribs (IR)	inconspicuous 0; prominent 1
7	Leaflet length (LL)		19	Fruit sequence color (FSC)	green/red = 2 0; green/red = 1 1; green/red = 1/2 2; red 3
8	Leaflet width (LW)		20	Seed number (SN)	
9	Leaf index (LI)		21	Seed length (SL)	
10	Leaf margin (LM)	obtuse serrate 0; serrulate 1	22	Seed width (SW)	
11	Leaf texture (LT)	paper 0; thick paper 1; membranous 2	23	Seed index (SI)	
12	Petiole length (PL)				
		

The formula of leaf, fruit, and seed indices were as follows: Leaf index = leaf length/leaf width; fruit index = fruit length/fruit width; seed index = seed length/seed width.

Phenotypic observations were carried out on twenty fruits, twenty leaves, twenty seeds, and twenty compound leaves per sample. For the qualitative traits of fruits and annual branches, all observations were made on the period of pericarp dehiscence. Fruits and annual branches were randomly collected from the canopy of each tree and brought back to the laboratory for measurement of quantitative traits of fruits, leaves and seeds. For seeds sampled from the sampled fruit. Leaves were sampled from the middle of the second or third pairs of compound leaves of the annual branches. To minimize environmental effects on all the phenotypes, data were collected across two years (2016–2017) for each sample [28,29].

### Statistical methods

R package (R version 3.2.5) was used to calculate the Shannon–Wiener index (*I*), distribution and frequency of qualitative traits, and maximum, minimum, average (X), standard deviation (SD), coefficient of variation (CV) of each quantitative trait, which was calculated as follows: CV = SD/X. In addition, R package was used to analyze the correlation between phenotypic traits and geographical environmental factors in order to explore the effects of geographical environmental factors on phenotypic traits. Each trait was also subject to one-way analysis of variance (ANOVA) at a significant level of *p* < 0.05 [30].

Principal component analysis (PCA) is commonly used to study patterns of variation in a set of interrelated traits through the identification of subsets, called factors, which are substantially correlated, simultaneously affecting these traits to a large extent [31]. The purpose of principle component analysis was to reduce the number of observed variables into a relatively smaller number of components [6]. Examination of the eigenvalue is required to judge whether a principal component (PC) is meaningful. If this number is >1.0, then theoretically, the corresponding PC provides more information than any single variable and may be considered a major factor [32]. In this paper, the principal component analysis performed after standardizing all traits using R package. Furthermore, eigenvalues and relative proportion of the variance explained by each trait were calculated [33].

R package also was used to cluster all data with Q-type and R-type clustering. Using different units of measure resulted in entirely different types of scales with unequal weights, thus the data were standardized. The method of standard deviation standardization was selected. In order to explore the global similarity of phenotypic traits among different populations, Q-type clustering was performed using the class average chain method and Euclidean distance. In order to explore the degree of correlation between the different traits, R-type clustering was performed. The clustering method and distance coefficient were similar to that of Q-type clustering.

## Results

### Analysis of phenotypic diversity

The Shannon–Wiener index (*I*) is an important index for evaluating the diversity of germplasm resources. Among the seven qualitative traits in this study, the *I* of fruit color (FC: 1.26), fruit sequence color (FSC: 1.25), annual branch color (ABC: 0.96), and compound petiole color (CPC: 0.89) were relatively high, while those of irregular ribs (IR: 0.55), leaf margin (LM: 0.55), and leaf texture (LT: 0.68) were relatively low. The results show that color traits play an important role in distinguishing different provenances of *E*. *japonica*, and evaluating its phenotypic diversity (Table 3).

**Table 3:** Distribution and frequency of qualitative traits in populations of *E*. *japonica*.

Trait	Shannon-Wiener (*I*)		Zunyi	Qingliu	Jianyang	Jianou	Wenzhou	Jiangshi	Taining	Dehua	JX	Total sample
CPC	0.89	0	8(88.89%)	4(40%)	3(100%)	2(66.67%)	3(60%)	12(92.31%)	5(100%)	3(50%)	0(0%)	40(59.70%)
		1	1(11.11%)	4(40%)	0(0%)	1(33.33%)	2(40%)	1(7.69%)	0(0%)	3(50%)	9(69.23%)	21(31.34%)
		2	0(0%)	1(10%)	0(0%)	0(0%)	0(0%)	0(0%)	0(0%)	0(0%)	4(30.77%)	5(7.46%)
		3	0(0%)	1(10%)	0(0%)	0(0%)	0(0%)	0(0%)	0(0%)	0(0%)	0(0%)	1(1.49%)
FC	1.26	0	0(0%)	0(0%)	1(33.33%)	0(0%)	0(0%)	0(0%)	2(40%)	0(0%)	1(7.69%)	4(5.70%)
		1	0(0%)	2(20%)	1(33.33%)	1(3334%)	0(0%)	7(53.85%)	0(0%)	3(50%)	6(46.15%)	17(25.37%)
		2	1(11.11%)	6(60%)	1(33.34%)	2(66.66%)	0(0%)	4(30.77%)	3(60%)	3(50%)	6(46.15%)	26(38.80%)
		3	8(88.89%)	2(20%)	0(0%)	0(0%)	5(100%)	2(15.38%)	0(0%)	3(50%)	0(0%)	20(29.85%)
FSC	1.25	0	0(0%)	2(20%)	2(66.66%)	0(0%)	2(40%)	1(7.69%)	1(20%)	0(0%)	5(38.46%)	13(19.40%)
		1	2(22.22%)	1(10%)	1(33.34%)	1(33.34%)	1(20%)	4(30.77%)	3(60%)	1(16.67%)	4(30.77%)	18(26.87%)
		2	5(55.56%)	5(50%)	0(0%)	2(66.66%)	2(40%)	8(61.54%)	1(20%)	2(33.33%)	4(30.77%)	29(43.28%)
		3	2(22.22%)	2(20%)	0(0%)	0(0%)	0(0%)	0(0%)	0(0%)	0(0%)	0(0%)	7(10.44%)
ABC	0.95	0	9(100%)	4(40%)	3(100%)	2(66.66%)	3(60%)	11(84.62%)	4(80%)	0(0%)	5(38.46%)	41(61.20%)
		1	0(0%)	3(30%)	0(0%)	1(33.34%)	2(40%)	2(15.38%)	0(0%)	3(50%)	8(61.54%)	19(28.36%)
		2	0(0%)	2(20%)	0(0%)	0(0%)	0(0%)	0(0%)	1(20%)	0(0%)	0(0%)	3(4.48%)
		3	0(0%)	1(10%)	0(0%)	0(0%)	0(0%)	0(0%)	0(0%)	3(50%)	0(0%)	4(5.97%)
LT	0.68	0	9(100%)	0(0%)	0(0%)	0(0%)	5(100%)	0(0%)	0(0%)	0(0%)	0(0%)	14(20.90%)
		1	0(0%)	0(0%)	0(0%)	0(0%)	0(0%)	0(0%)	0(0%)	3(50%)	1(7.69%)	4(5.97%)
		2	0(0%)	10(100%)	3(100%)	3(100%)	0(0%)	13(100%)	5(100%)	3(50%)	12(92.31%)	49(73.13%)
LM	0.55	0	0(0%)	10(100%)	3(100%)	3(100%)	0(0%)	13(100%)	5(100%)	3(50%)	12(92.31%)	49(73.13%)
		1	9(100%)	0(0%)	0(0%)	0(0%)	5(100%)	0(0%)	0(0%)	3(50%)	1(7.69%)	18(26.87%)
IR	0.55	0	0(0%)	10(100%)	3(100%)	3(100%)	0(0%)	13(100%)	5(100%)	3(50%)	12(92.31%)	49(73.13%)
		1	9(100%)	0(0%)	0(0%)	0(0%)	5(100%)	0(0%)	0(0%)	3(50%)	1(7.69%)	18(26.87%)

For color traits, the color of fruit (FC) and fruit sequence (FSC) mainly belonged to 2-type, with distribution frequencies of 38.80% and 43.28%, respectively, followed by 1-type. The color of compound petiole (CPC) was mainly 0-type. Furthermore, compound petiole color (CPC) was greenish, while fruit color (FC) and fruit sequence color (FSC) were reddish. In addition, leaf texture was mainly membrane (73.13%), followed by paper (20.90%), and thick paper (5.97%) (Table 3).

The coefficient of variation (CV) for quantitative traits indicates phenotypic traits’ degree of dispersion. Thus, the larger the coefficient of variation, the greater the dispersion values of the measured traits. The average coefficient of variation of different quantitative traits of *E*. *japonica* were as follows: Petiole length (PL: 23.95%) > seed number (SN: 21.54%) > leaflet circumference (LC: 18.30%) > leaflet area (LA: 17.85%) > pericarp thickness (PT: 15.82%) > fruit length (FL: 14.05%) > compound leaf length (CLL: 13%. 91%) > fruit shape index (FS: 12.75%) > leaflet width (LW: 12.10%) > leaflet number (LN: 11.42%) > leaf shape index (LS: 10.88%) > seed shape index (SS: 9.96%) > leaflet length (LL: 9.47%) > seed width (SW: 8.57%) > fruit width (FW: 8.43%) > seed length (SL: 5.79%). It is therefore obvious that the coefficient of seed traits’ variation were relatively backward, indicating that seed variation was small. Comparing the average coefficient of variation of eight compound leaf traits, four fruit and seed traits, showed that the average coefficient of variation of compound leaf traits (14.74%) was higher than that of fruit traits (12.77%) and seed traits (11.47%), indicating that seed traits were relatively stable during the process of evolution, while rich variation in compound leaf and fruit traits (Table 4).

**Table 4:** The coefficient of variation (CV%) on Quantitative traits in populations of *E*. *japonica*.

CV(%)	Zunyi	Qingliu	Jianyang	Jianou	Wenzhou	Jiangshi	TainingN	Dehua	Jiangxi	average
LA	24.79	18.68	5.53	11.86	11.49	24.75	20.13	18.03	25.38	17.85
LC	12.56	8.69	5.67	6.96	9.96	11.90	13.41	83.13	12.38	18.30
LL	14.27	8.82	7.99	7.75	3.75	11.08	13.19	8.82	9.55	9.47
LW	13.12	12.15	6.20	13.94	8.57	14.68	12.72	12.02	15.53	12.10
LI	9.96	11.44	13.44	14.64	9.86	10.51	6.36	11.80	9.93	10.88
CLL	11.37	12.65	21.33	10.90	18.04	13.98	19.84	12.78	4.29	13.91
LN	9.79	10.27	14.15	13.06	12.99	8.66	15.51	13.17	5.23	11.43
PL	36.02	10.30	24.41	17.43	20.42	24.32	9.90	44.41	28.34	23.95
FL	32.93	10.81	24.00	7.07	6.98	15.69	12.83	7.55	8.63	14.05
FW	7.42	9.07	3.90	9.87	5.93	7.41	3.63	12.55	16.14	8.43
FI	30.07	5.77	19.72	7.50	7.75	8.92	10.38	13.37	11.25	12.75
PT	14.61	19.08	13.17	9.01	9.92	17.46	9.52	28.19	21.47	15.83
SN	24.50	23.49	17.52	22.17	9.42	24.61	27.90	16.44	27.79	21.54
SL	5.64	6.39	8.54	8.76	4.03	7.50	2.36	4.77	4.10	5.79
SW	4.52	9.75	3.15	9.79	3.92	5.90	29.16	5.33	5.67	8.58
SI	5.23	6.66	7.83	4.59	2.14	4.73	45.37	9.85	3.29	9.97
average	16.05	11.50	12.28	10.96	9.07	13.26	15.76	18.89	13.06	—

In order to explore the contribution of individual traits to phenotypic variation, we have principal component analysis for all quantitative traits (Table 5). Using an eigenvalue greater than one as a measure of PC significance, five PCs accounted for 74.23% of the total variability in the data, which retained the majority of the information represented by the original factor. Due to the substantially high correlation between PC1 and leaflet area (LA: 0.912), leaflet length (LL: 0.861), leaflet width (LW: 0.806), and pericarp thickness (PT: 0.804), the first principal component represented these inter-correlated traits to a large extent and was therefore the most important genetic factor, accounting for 28.96% of the total variance. Thus, PC1 mainly reflects compound leaf and fruit characters. PC2 correlated positively with leaf index (LI: 0.629) and leaflet number (LN: 0.587), and negatively with seed width (SW: - 0.716) and seed length (SL: -0.645). Therefore, PC2 represented leaf and seed traits, accounting for 16.04% of the total variance. PC3 correlated positively with fruit width (FW: 0.701) and fruit index (FI: 0.801), which represent fruit characters. PC4 correlated positively with seed index (SI: 0.614) and petiole length (PL: 0.518), and PC5 correlated positively with petiole length (PL: 0.597). It is seen that PC4 and PC5 mainly reflected leaf characters (Table 6). In summary, we found that the leaf and fruit traits contributed the most to the phenotypic variation of *E*. *japonica*.

**Table 5:** Eigenvalues, proportion of total variability and correlation between the original variables and the first five principal components (PCs).

Phenotypic traits	Characteristic load quantity					Total load
	PC1	PC2	PC3	PC4	PC5	
LA	0.91	-0.17	0.05	0.17	-0.03	0.89
LC	0.38	0.10	-0.13	-0.32	0.39	0.43
LL	0.86	0.15	0.05	0.15	0.20	0.83
LW	0.81	-0.36	0.08	0.33	-0.19	0.93
LI	-0.17	0.64	-0.07	-0.35	0.49	0.80
CLL	0.73	0.35	-0.18	-0.25	-0.11	0.77
LN	0.32	0.59	-0.06	-0.09	0.01	0.46
PL	0.16	0.23	0.06	0.52	0.60	0.70
FL	0.52	0.25	0.70	-0.06	-0.13	0.84
FW	0.77	-0.17	-0.02	-0.24	0.05	0.68
FI	-0.05	0.43	0.80	0.12	-0.18	0.87
PT	0.80	-0.04	-0.15	-0.35	-0.07	0.80
SN	-0.09	0.39	0.46	0.32	0.11	0.48
SL	0.15	-0.65	0.03	0.34	0.35	0.68
SW	-0.06	-0.72	0.48	-0.29	0.32	0.93
SI	0.18	0.32	-0.52	0.61	-0.10	0.78
Eigenvalue	4.64	2.57	1.93	1.60	1.14	
Contribution rate (%)	28.96	16.06	12.06	10.00	7.15	
Cumulative contribution rate (%)	28.96	45.03	57.09	67.08	74.23	

**Table 6:** Correlation coefficient between phenotypic traits and geographic factors of *E*. *japonica*.

Phenotypic traits	Geographic information		
	Longitude	Latitude	Altitude /m
LA	0.044	0.269	-0.221
LC	-0.167	0.283	-0.227
LL	0.043	0.146	-0.505
LW	0.133	0.24	-0.076
LI	-0.402	0.089	-0.288
CLL	0.01	-0.244	-0.578
LN	-0.219	-0.392	0.127
PL	0.078	0.089	0.173
FL	0.285	-0.492	0.11
FW	-0.105	0.208	-0.185
FI	0.325	-0.565	0.185
PT	0.421	-0.237	-0.464
SN	0.38	-0.222	0.412
SL	0.255	0.313	0.139
SW	0.264	-0.07	0.448
SI	-0.041	0.376	-0.357
CPC	0.171	-0.447	0.587
FC	0.565	-0.224	-0.932**
FSC	0.302	-0.36	-0.319
LT	0.174	-0.332	0.670*
LM	-0.174	0.332	-0.670*
IR	-0.174	0.332	-0.670*
—	4.732	6.262	8.343

Note

* the difference is significant

** the difference is extremely significant.

**Fig 2:**
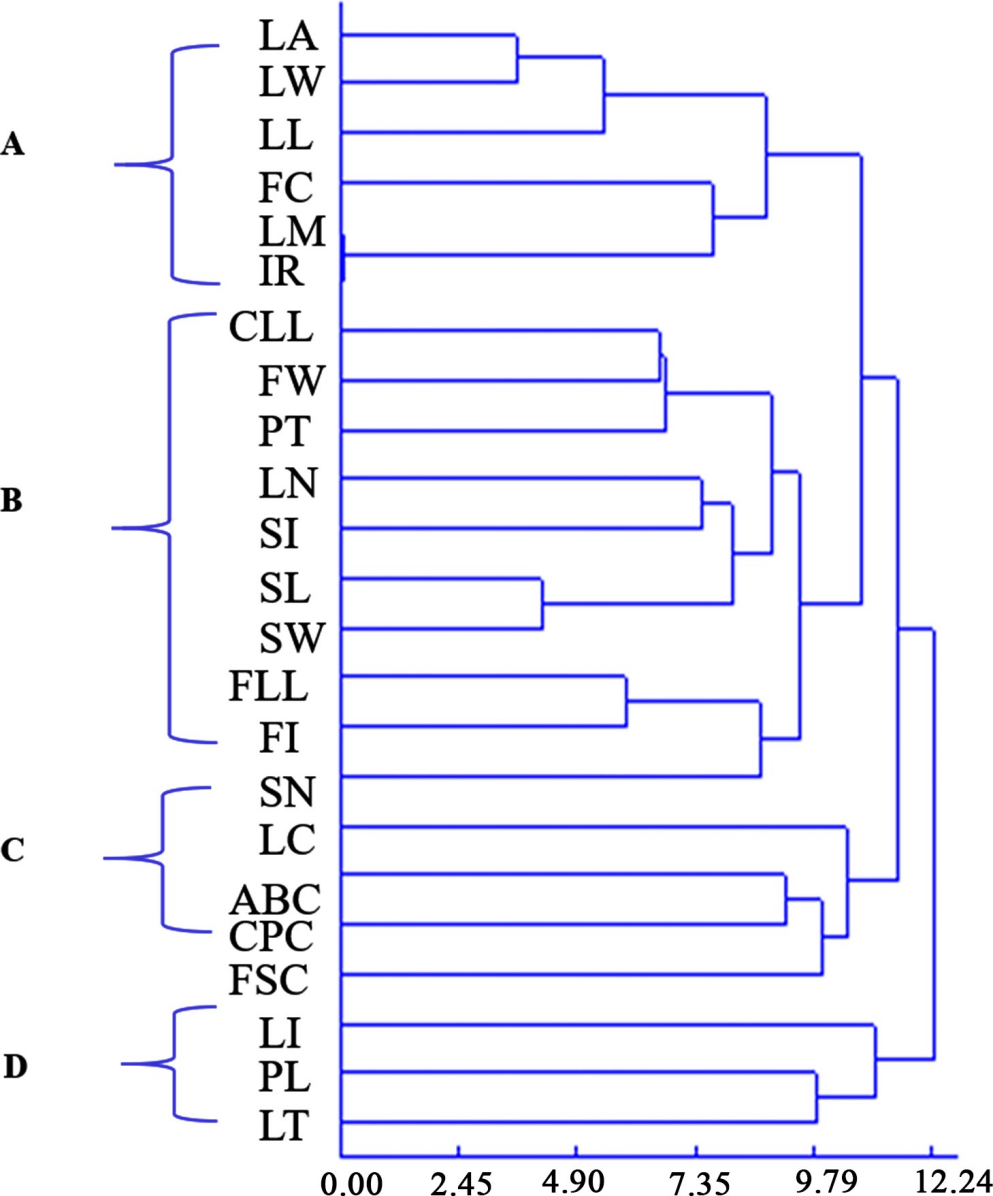
R dendrogram based on morphologic data. Each branch represents a trait.

Although principal component analysis (PCA) revealed which traits played an important role in phenotypic variation, it could not identify the relationship between phenotypic traits. Thus, the R-type cluster of 23 traits was carried out. As shown in Fig 2, the clustering result was clearly divided into five groups. Group A contained leaflet area (LA), leaflet width (LW), leaflet length (LW), fruit color (FC), leaf margin (LM), and irregular ribs (IR), which mainly reflected leaflet characters. Group B contained compound leaf length (CLL), fruit width (FW), pericarp thickness (PT), leaflet number (LN), seed shape index (SI), seed length (SL), seed width (SW), fruit length (FL), fruit shape index (FI), and seed number (SN), which mainly reflected fruit and seed characters. Group C contained leaflet circumference (LC), annual branch color (ABC), compound petiole color (CPC), and fruit sequence color, which mainly reflected color characters. Group D only contained the leaf shape index (LI), and Group E contained petiole length and leaf texture. Groups D and E also reflected leaf characters. The results of the R-type clustering reflected the correlation between some phenotypic traits, such as the color of annual branch, compound petiole, and fruit sequence. Conversely, some of the traits that appeared in proximity on the dendogram were not relevant, such as leaf margin and irregular ribs, whose genetic background requires further study.

Our sampling range is wide, covering 4 provinces, from low altitude to high altitude. Therefore, we explore whether the phenotypic variation is related to altitude, latitude and longitude. Comparing the correlation coefficients between geographic factors and phenotypic traits showed that the altitude was the highest (8.343), followed by latitude (6.262), and longitude was the lowest (4.732) (Table 6), indicating that the effect of altitude on the phenotypic variation of *E*. *japonica* was greater than that of latitude and longitude. Furthermore, we identified a significant negative correlation between altitude, fruit color (FC) and leaf margin (LM), as well as a significant positive correlation with leaf texture (LT) and irregular rib (IR).

### Analysis of phenotypic diversity within and between populations

The population’s phenotypic diversity positively correlates with the coefficient of variation. The higher the coefficient of variation, the richer the phenotypic diversity. Comparing the coefficients of variation for nine populations showed that Dehua was the highest at 18.89%, followed by Zunyi, and the smallest was Wenzhou (Table 4).

The results from the analysis of 16 quantitative traits in different populations are shown in Tables 7 and 8. The seed index (SI), seed width (SW), leaflet area (LA), leaflet stem length (PL), and leaflet circumference (LC) of *E*. *japonica* reached a significant level within populations. Among 16 quantitative traits, except for fruit length (FL), the differences in other traits were significant among populations. This implied that the variation in quantitative traits among populations was much greater than within populations.

**Table 7:** ANOVA analysis on compound leaf traits in populations of *E*. *japonica*.

Population		Zunyi	Qingliu	Jianyang	Jianou	Wenzhou	Jiangshi	TainingN	Dehua	Jiangxi	Interpopulation F value	Intrapopulation F value
LA	Range	1157.11~2645.36	875.15~1707.90	1033.63~1173.16	739.57~1049.77	1059.48~1454.26	755.18~1544.19	917.68~1377.13	702.78~1158.81	649.91~1781.22	9.43**	2.55*
	X+SD	1809.22±448.58	1217.63±227.51	1091.88±60.34	904.82±107.29	1313.65±150.91	1039.70±257.34	1103.59±222.11	931.95±168.02	1089.83±276.56		
LL	Range	168.58~248.47	126.55~174.34	139.94~160.46	124.69~151.02	141.01~180.36	116.42~168.43	123.68~168.81	129.68~556.78	119.35~190.16	2.05*	6.98**
	X+SD	194.28±24.40	146.61±12.74	148.49±8.43	137.02±9.54	154.72±15.41	135.34±16.10	141.75±19.00	206.54±171.71	141.28±17.49		
LL	Range	65.70~102.10	57.70~76.79	59.90~72.40	55.00~66.40	59.10~65.00	53.00~77.30	54.50~76.00	51.60~65.71	55.20~79.70	6.58**	1.28
	X+SD	79.30±11.32	65.75±5.80	66.03±5.28	60.21±4.67	61.40±2.31	61.23±6.79	64.40±8.50	59.68±5.27	63.45±6.06		
LW	Range	26.53~41.56	23.26~34.06	25.50~29.75	21.61~31.66	28.09~34.75	21.20~32.21	24.00~31.85	20.38~28.02	20.58~36.56	6.67**	0.87
	X+SD	34.95±4.58	28.92±3.51	28.03±1.74	26.48±3.69	33.13±2.84	26.01±3.82	27.75±3.53	24.50±2.94	26.84±4.17		
LI	Range	1.80~2.62	1.79~2.85	2.09~2.77	1.80~2.69	1.72~2.18	1.95~2.89	2.13~2.47	2.13~2.80	2.05~2.75	2.44*	1.02
	X+SD	2.28±0.23	2.30±0.26	2.37±0.32	2.31±0.34	1.87±0.18	2.38±0.25	2.32±0.15	2.46±0.29	2.39±0.24		
CLL	Range	14.96~21.80	10.75~16.54	8.65~15.57	9.57~12.75	7.98~12.78	10.08~17.26	9.66~15.36	9.41~12.85	10.29~11.88	14.09**	1.99
	X+SD	18.11±2.06	13.10±1.66	12.27±2.62	10.96±1.19	11.25±2.03	12.98±1.81	11.81±2.34	11.05±1.41	11.35±0.49		
LN	Range	6.60~9.40	6.56~9.10	6.60~9.30	6.10~8.67	5.00~7.00	6.40~8.38	6.00~9.10	6.17~8.50	7.50~9.00	5.47**	1.48
	X+SD	8.36±0.82	7.67±0.79	8.00±1.13	7.60±0.99	6.08±0.79	7.39±0.64	7.30±1.13	7.47±0.98	8.51±0.45		
PL	Range	3.26~8.84	5.65~7.70	5.38~10.21	4.78~7.42	3.39~5.49	3.56~8.67	7.23~9.43	2.30~5.86	2.90~7.73	6.02**	2.10*
	X+SD	5.20±1.87	6.71±0.69	7.29±1.78	5.83±1.02	4.53±0.92	5.95±1.45	8.12±0.80	3.95±1.75	4.72±1.34		

Note

* the difference is significant

** the difference is extremely significant.

**Table 8:** ANOVA analysis on fruit and seed traits in populations of *E*. *japonica*.

Population		Zunyi	Qingliu	Jianyang	Jianou	Wenzhou	Jiangshi	TainingN	Dehua	Jiangxi	Interpopulation F value	Intrapopulation F value
FI	Range	1.18~2.70	1.52~1.82	1.55~2.59	1.58~1.95	1.25~1.50	1.41~1.86	1.38~1.76	1.46~1.92	1.26~1.82	2.13*	1.33
	X+SD	1.51±0.45	1.64±0.09	1.87±0.37	1.73±0.13	1.38±0.11	1.61±0.14	1.51±0.16	1.61±0.22	1.66±0.19		
PT	Range	1.78~3.05	1.13~1.98	0.84~1.21	0.94~1.24	1.19~1.50	0.98~1.87	0.99~1.25	0.71~1.36	1.03~1.89	23.73**	0.93
	X+SD	2.30±0.34	1.36±0.26	1.00±0.13	1.05±0.09	1.28±0.13	1.24±0.22	1.08±0.10	1.03±0.29	1.28±0.28		
SN	Range	1.10~2.10	1.30~2.60	1.70~2.56	1.70~3.00	1.00~1.20	1.20~2.80	1.00~2.00	1.00~1.50	1.10~2.57	4.49**	1.22
	X+SD	1.43±0.35	1.74±0.41	1.94±0.34	2.19±0.48	1.07±0.10	1.66±0.41	1.42±0.40	1.25±0.21	1.79±0.50		
SL	Range	4.30~5.18	4.45~5.47	3.81~4.94	4.42~5.39	4.91~5.36	4.03~5.05	5.01~5.27	4.18~4.65	4.44~5.37	5.56**	1.82
	X+SD	4.69±0.26	4.89±0.31	4.43±0.38	4.75±0.42	5.15±0.21	4.53±0.34	5.14±0.12	4.46±0.21	4.94±0.20		
SW	Range	4.05~4.55	4.18~5.55	3.94~4.28	4.05~5.30	4.72~5.14	3.79~4.77	2.02~4.96	4.15~4.73	4.08~4.99	3.34**	5.30**
	X+SD	4.28±0.19	4.72±0.46	4.08±0.13	4.49±0.44	4.95±0.19	4.16±0.25	4.16±1.21	4.47±0.24	4.57±0.26		
SI	Range	1.01~1.18	0.89~1.13	0.97~1.21	1.00~1.13	1.01~1.07	1.03~1.21	1.06~2.50	0.88~1.12	1.02~1.13	2.23*	9.67**
	X+SD	1.10±0.06	1.04±0.07	1.09±0.08	1.06±0.05	1.04±0.02	1.09±0.05	1.38±0.63	1.00±0.10	1.08±0.04		
FL	Range	10.51~23.96	10.14~13.82	9.33~16.80	10.37~12.71	8.59~10.11	9.08~15.51	8.96~11.98	8.83~10.53	9.69~13.72	1.68	1.72
	X+SD	12.87±4.24	11.60±1.25	11.40±2.74	11.12±0.79	9.55±0.67	11.34±1.78	10.02±1.29	9.92±0.75	10.99±0.95		

Note

* the difference is significant

** the difference is extremely significant.

The F value of leaflet circumference (LC), fruit length (FL), and seed width (SW) were lower among populations than within populations. Furthermore, the F value of the remaining quantitative traits were higher between populations than within populations, further suggesting the presence of abundant phenotypes among populations. In compound leaf traits, except leaflet circumference (LC), the F value among populations of the remaining traits were more than double compared to within populations’ values. In fruit traits, the F value among populations of pericarp thickness (23.73) was 25.51 times that within populations; followed by fruit width (FW), which was 5.55 times that within populations. In seed traits, the F value of seed index (SI) and seed width (SW) were the highest within populations, 9.67 and 5.30, respectively. Therefore, it is obvious that compound leaf and fruit traits’ variations were rich among populations, while seed traits’ variations were relatively stable (Tables 7 and 8).

Multiple comparative analyses of compound leaf traits among populations showed that (Table 7) the mean value of leaflet area (LA), leaflet length (LL), leaflet width (LW), and compound leaf length (CLL) of Zunyi were the highest, followed by Wenzhou, while Dehua had the lowest values. Interestingly, the leaf index (LI) of Wenzhou was the lowest (1.87 ± 0.18), while Dehua had the maximum leaf index value (2.46 ±0.29). Therefore, Zunyi and Wenzhou populations were defined as large-leaf *E*. *japonica*, and Dehua as small-leaf *E*. *japonica*. The mean values of fruit width (FW), fruit length (FL), and pericarp thickness (PT) of the Zunyi population were the highest, while Jianyang population exhibited the lowest mean value of fruit width (FW), pericarp thickness (PT), and fruit shape index (FS) (Table 8). Finally, variable ranges of seed number (SN), seed length (SL), seed width (SW), and seed shape index (SS) were 1.00–3.00, 3.81–5.47, 2.20–5.55, and 0.88–1.21, respectively, among which the variation range of seed width was the largest (Table 8).

### Cluster analysis between different populations

**Fig 3:**
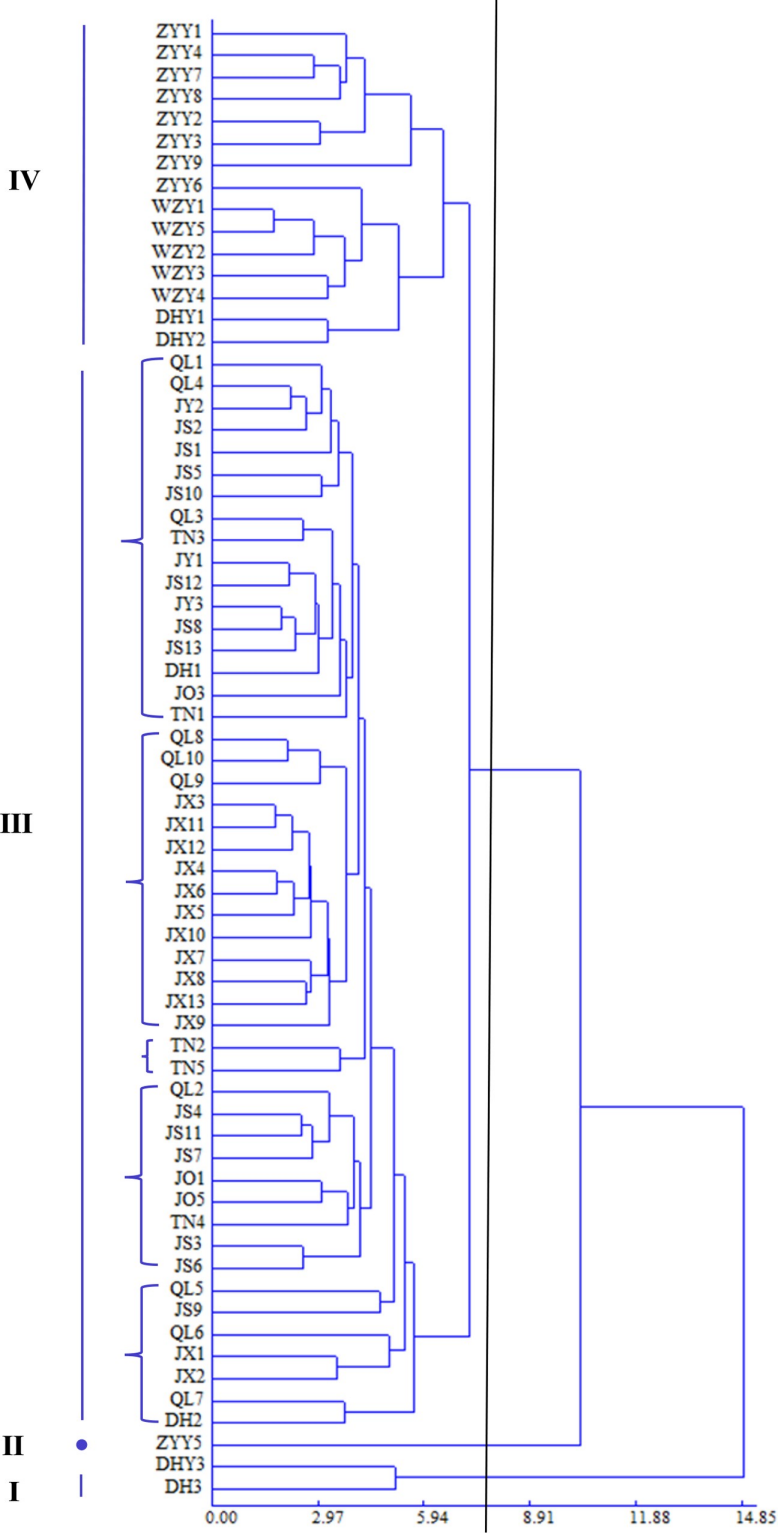
Q dendrogram of *E*. *japonica* based on morphologic data. Each branch represents a sample.

The dendogram obtained from the Q-type clustering performed on the 23 phenotypic traits is shown in Fig 3. Using the value 7.42, i.e., approximately 50% of the standardized maximum distance for the separation of groups, as a dendrogram cutting point criteria [34,35], four groups were distinguished (Fig 3). DH3 and DHY3 comprised group Ⅰ, and group II was only sample ZYY5. Fifteen samples comprised group Ⅳ, which contained the remaining samples of Zunyi, all samples of Wenzhou, as well as DHY1 and DHY2 of Dehua. Group III included 49 samples from the Zhejiang and Fujian populations, and was further subdivided into five subgroups. The first subgroup included JX2, JX9 of JX, and QL5 – 7of Qingliu. The second subgroup included some samples of Jiangshi, Taining, and Jianou. TN2 and TN3 were grouped together in the third subgroup. The fourth subgroup mainly included most samples from Jiangxi and Qingliu. The fifth subgroup contained 17samples from the Fujian province.

## Discussion

It is known that endemic woody species have low genetic variation, while distributed and widespread species maintain high variation [35–38]. In this study, we focused on the natural *E*. *japonica* populations, and our sampling range covers four provinces, from low altitude to high altitude (100m ~1000m). Studied on 23 traits of nine populations showed that there were abundant phenotypic variations in leaf and fruit of *E*. *japonica*, and principal component analysis also showed that the leaf and fruit variation were important components of the total variability, indicating that the variation of leaflet and fruit traits were regarded as an important evaluation index in the diversity evaluation of *E*. *japonica* from different provenances, and leaves and fruits are important genetic resources for breeding of *E*. *japonica*.

In R-type cluster, the color traits clustered into group C, leaf margin and irregular ribs were closely together. And altitude had a great influence on phenotypic characters. Interestingly, there are consistent with our observations in the field. Field investigation showed that the higher the altitude, the redder the fruit color; and the reddish fruit color, the reddish color of fruit sequence, the color of annual branch and the color of compound petiole. we also found that *E*. *japonica* with red fruit, epidermis ribs prominent, leafy papery or thick papery, margin serrulate were present in high-altitude areas (300m~1000m), while *E*. *japonica* with fruit epidermis rib inconspicuous, leaf membranous, and margin obtuse serrate were present in low-altitude areas (100m~500m).

Diversity variation among populations is an important component of species diversity, reflecting population adaptation in different environments, where the magnitude of variation indicates to some extent the species ability to adapt to different environments [39,40]. In this study, the variation in phenotypic traits among populations was much greater than within populations. Furthermore, Zunyi and Wenzhou populations were defined as large-leaf *E*. *japonica*, and Dehua as small-leaf *E*. *japonica*. Analysis of the average coefficient of variation for each population showed that the coefficient of variation for Dehua and Zunyi populations were the highest, Wenzhou population was smallest. It may be that Dehua and Zunyi populations are located in forests and nature reserves and their habitats with little human interference and good habitat protection.

The evolution of life is driven by natural selection acting on phenotypic trait variation among individuals in a population [41]. The variations in these traits are genetically controlled but could be highly influenced by environment conditions [42]. In this study, *E*. *japonica* from different provenances clustered into four groups, according to the similarities in phenotypic traits. In combination with long-term field observation, these four groups clearly formed deciduous *E*. *japonica* and evergreen *E*. *japonica*, and the qualitative and quantitative traits of the two types were quite different. The deciduous *E*. *japonica* composed of the population of Wenzhou, Zunyi, and Dehua (DHY1, DHY2, DHY3) were located in the mountainous area at 300 m above sea level. The characteristics of these populations are: deciduous trees or shrubs with leafy papery or thick papery, margin serrulate, epidermis ribs prominent, red exocarp; florescence is from April to May, fruit is from July to December, and fruit discoloration is in June, leaf color turns yellow in autumn. Jiangxi population and the populations in Fujian Province constituted evergreen *E*. *japonica*, located in the mountainous area with low altitude (≤500m). The characters of these populations are: evergreen trees or shrubs with leaf membranous, margin obtuse serrate, fruit epidermis rib inconspicuous, red and green exocarp; the florescence is from May to June, fruit is from September to March, and fruit discoloration is in August. We suggest that the *E*. *japonica* populations at similar elevations are in similar ecological conditions, and in the long-term evolution process form similar adaptation matrices for similar environments, resulting in phenotypic similarities.

## Conclusion

In this study, we reveal considerable phenotypical diversity among nine populations of *E*. *japonica* from four provinces. Variations in leaf and fruit traits were abundant, indicating that improvement in *E*. *japonica* is broad, and the rich phenotypic variation provided a material basis for germplasm resources and diversity. Principal component analysis also verified that leaflet and fruit traits had a great contribution to the principal components. In addition, the correlation analysis revealed a significant difference in elevation and fruit color, irregular ribs, leaf margin, and leaf texture. The variation in phenotypic traits among populations was much greater than within populations. Furthermore, Zunyi and Wenzhou populations were defined as large-leaf *E*. *japonica*, and Dehua population as small-leaf *E*. *japonica*. Dehua and Zunyi populations had the highest coefficients of variation, and WZ had the lowest—possibly due to habitat destruction. Q-type clustering grouped the samples according to their phenotypic similarities. All samples clustered into four groups, basically distinguishing *E*. *japonica* of different provenances. Combining the clustering results and field surveys, showed that the clustering result clearly formed t deciduous *E*. *japonica* and evergreen *E*. *japonica*. One is the deciduous *E*. *japonica* at high altitudes, and the other is the evergreen *E*. *japonica* at low altitudes. The inventory of *E*. *japonica* based on phenotypic descriptions is the first study to give insight into the extent of phenotypic diversity and is of great importance for planning genetic resources preservation strategy and establishing collections.
